# Serum progranulin/tumor necrosis factor-α ratio as independent predictor of systolic blood pressure in overweight hypertensive patients: a cross-sectional study

**DOI:** 10.1186/s43044-020-00063-3

**Published:** 2020-05-18

**Authors:** Jasmine Kaur, Supriya Mukheja, Sudhir Varma, Harpreet Singh Kalra, Bipanjeet Singh Khosa, Kanchan Vohra

**Affiliations:** 1 Department of Pharmaceutical Sciences and Drug Research, Punjabi University, Patiala, India; 2 Sadbhavna Medical & Heart Institute, Patiala, India; 3 A. P. Jain Civil Hospital, Rajpura, Patiala, India

**Keywords:** Progranulin, Tumor necrosis factor-α, Progranulin/tumor necrosis factor-α ratio, Systolic-diastolic hypertension, Isolated systolic hypertension, Independent predictor, Systolic blood pressure

## Abstract

**Background:**

Vascular inflammation plays a key role in the progression of hypertension. Progranulin (PGRN), an anti-inflammatory growth factor, mediated inhibition of tumor necrosis factor-α (TNF-α), a pleiotropic cytokine, activity has been well-established. Despite the role of chronic low-grade inflammation in hypertension, serum levels of PGRN and PGRN/TNF-α ratio and, their association with systolic and diastolic blood pressure has not been determined in hypertensive patients till now. This study aims to find and correlate the serum levels of pro-inflammatory cytokine (TNF-α), anti-inflammatory growth factor (PGRN), and PGRN/TNF-α ratio with the blood pressure in systolic-diastolic hypertension (SDH) and isolated systolic hypertension (ISH) patients.

**Results:**

A cross-sectional study was conducted on SDH patients (mean age, 52.95 ± 12.6 years; male/female (M/F) number = 15/10) and ISH patients (mean age, 55.80 ± 9.40 years; M/F number = 12/13) (*n* = 25 each). Twenty-five age and body mass index (BMI)-matched healthy subjects (mean age, 56.00 ± 8.55 years; male/female number = 11/14) were considered as control. All patients and healthy subjects were overweight (BMI, 25–30 kg/m^2^). Overnight fasting blood samples of subjects were taken and levels of PGRN and TNF-α were measured using ELISA diagnostic kits. PGRN and TNF-α levels were found significantly high, whereas PGRN/TNF ratio was found very low, in SDH and ISH patients as compared to healthy subjects. Reduced PGRN/TNF-α ratio and pulse pressure were found as independent predictors of SBP both in SDH and ISH patients.

**Conclusions:**

Findings of elevated PGRN levels in response to raised TNF-α levels depict the counter regulation by PGRN to neutralize TNF-α. Findings of reduced PGRN/TNF ratio, and it being an independent predictor of SBP, ascertain the key role of imbalance in pro- and anti-inflammatory environment in hypertension. Thus, it strengthens the cross-link between the concept of immunity–adiposity–inflammation–blood pressure¸ a vicious network. Further, this cross-link of SBP and progranulin must be explored in longitudinal studies. New researches should be focused not only on impact of pro-inflammatory environment rather to find on a balance between pro- and anti-inflammatory status, so that new target sites could be explored for therapeutic management of hypertension.

## Background

Hypertension globally accounts for 9.4 million deaths worldwide every year (WHO, 2015). Vascular inflammation plays a key role in the progression of essential hypertension [1]. Progranulin (PGRN), also known as granulin precursor 88/platelet cell (GP88/PC) derived factor, is a pleiotropic growth factor and one of the major anti-inflammatory molecules that plays an important part in the maintenance and regulation of the homeostatic dynamics in normal tissue development, proliferation, regeneration, and the host defense response [2]. PGRN is abundantly expressed in epithelial cells, immune cells, neurons and chondrocytes [3–5]. PGRN-mediated inhibition of tumor necrosis factor-alpha (TNF-α), a highly pleiotropic cytokine, activity has been well-established [6–8]. Serum PGRN levels has been estimated in various diseases which are characterized by presence of chronic low-grade inflammation such as atherosclerosis [9], neuro-degenerative diseases [10], breast cancer [11], diabetes [12], and metabolic syndrome [13]. Despite of the role of chronic inflammation in hypertension, novel anti-inflammatory marker serum PGRN levels and serum PGRN/TNF-α ratio had not been yet estimated in hypertensive patients and their association with systolic blood pressure (SBP) and diastolic blood pressure (DBP) is not explored.

## Methods

A prospective, cross-sectional study was conducted in hypertensive patients. The study protocol was approved (approval number 263/DLS/HG) by human institutional ethics committee (IEC) of the academic institute and was performed in accordance with the code of Good Clinical Practice and followed STROBE guidelines for observational studies. All patients provided written informed consent to participate after a full explanation of the study. Hypertensive patients visiting the hospital for their regular checkup/new diagnosis/or their ongoing treatment for hypertension (systolic-diastolic hypertension or isolated systolic hypertension) during a period of 8 months (March 2015 to October 2015) were assessed for eligibility (inclusion/exclusion) criteria of the study. Simple random sampling design was selected for inclusion of the subjects to exclude bias in selection of patients. Overall 80 patients were screened for hypertension and isolated systolic hypertension for eligibility criteria. Finally, 25 patients (age ≥ 18 years) of isolated systolic hypertension (ISH), defined as SBP ≥ 140 mmHg and DBP ≤ 90 mm Hg; and 25 patients of systolic-diastolic hypertension (SDH) defined as SBP ≥ 120 mmHg and DBP ≥ 90 mmHg according to the Eighth Joint National Committee (JNC)-VIII guidelines for hypertension [14] were recruited. Twenty-five age and body mass index (BMI)- matched healthy subjects were considered as control. Patients were excluded if having any other concomitant chronic inflammatory disease; history of type 1 and type 2 diabetes, continuous use of systemic steroids and NSAIDs; history of any concurrently present other major cardiovascular and metabolic disorders (congestive heart failure, stroke, ischemia, arrhythmia, dyslipidemia, thyroid disorders etc.); alcoholic and smokers; and pregnant and lactating women.

Clinical evaluations included the demographic profile and serum biomarkers. Overnight fasting blood samples of the patients and healthy subjects were withdrawn, serum was separated out and stored at – 40 °C till further analysis. The blood pressure was measured using manual mercurial sphygmomanometer on the same day just before withdrawing blood sample. Body mass index of subjects was calculated as weight in kilogram/square meter height [15]. Serum progranulin and TNF-α levels were measured using ELISA diagnostic kits (Krishgen Biosystems, India). Main outcome was to find serum PGRN level, TNF-α level, and the PGRN/TNF-α ratio to evaluate the pro- and anti-inflammatory environment of patients [16] and whether these can act as independent predictors of blood pressure.

Results were expressed as mean ± standard deviation (SD). One-way analysis of variance (ANOVA) test was used to compare the variables between the groups. The correlation between various clinical variables was examined by Pearson’s univariate correlation analysis. Multiple linear regression was used to reveal dependency of one variable on the other. All data were analyzed using Sigma Stat 3.5. Statistical significance was accepted at *p* ≤ 0.05.

Samples size was determined by considering serum progranulin level as primary marker for predicting blood pressure. Serum progranulin levels have been reported as 40.1 ± 8.7 ng/ml in health subjects by Yamamoto et al. 2014 [16]. Based on this finding, 25% elevated level of serum progranulin in hypertensives than healthy subjects were considered significant. Thus, to detect a difference of 25% at 95% power, a sample size of 25 subjects/group was required.

## Results

**Fig. 1:**
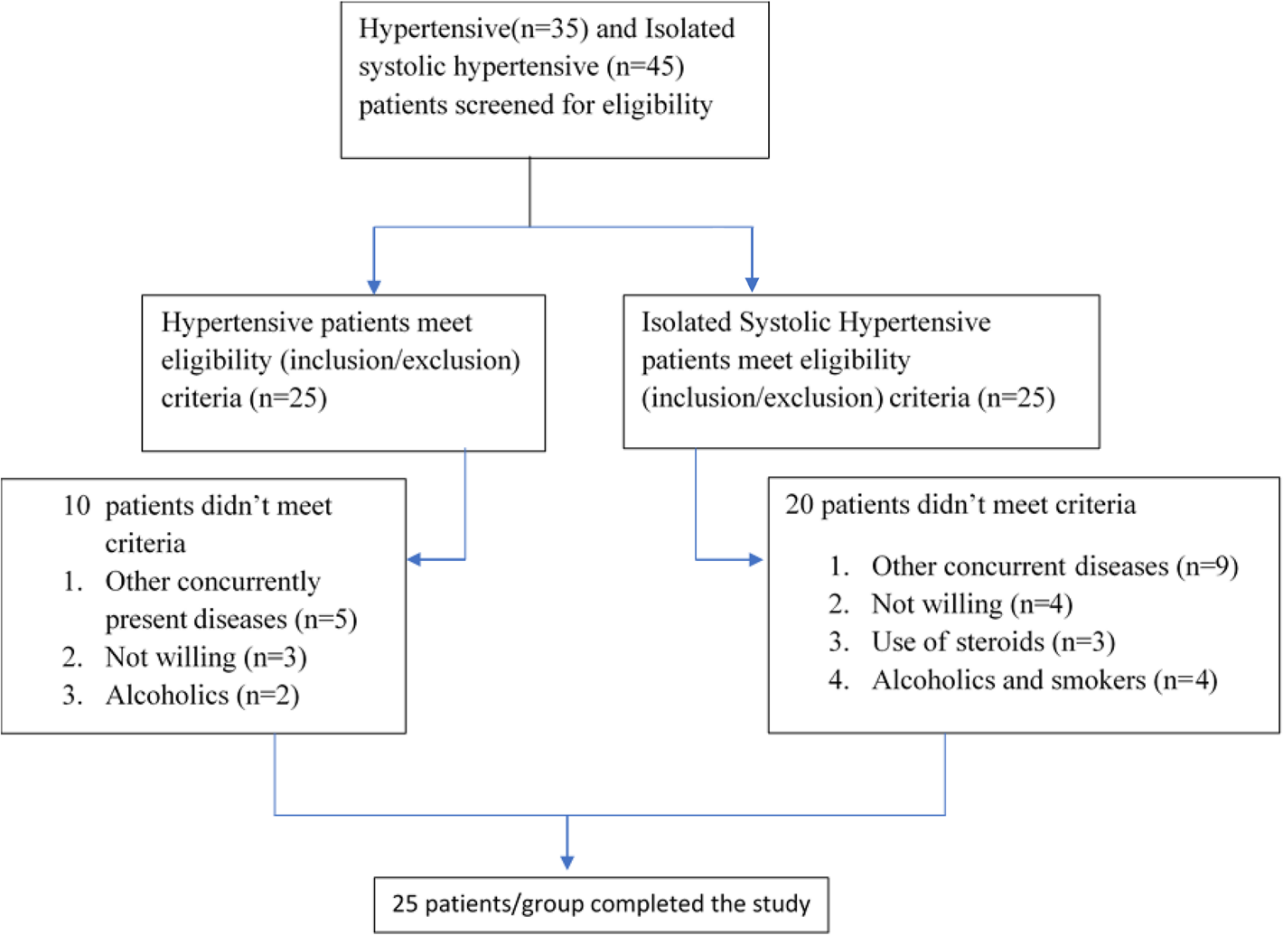
Selection criteria of study participants

The distribution of demographic, biochemical and hemodynamic characteristics are presented in Table 1. Figure 1 shows the flow chart of patients’ inclusion in the study. All the eligible patients as per inclusion/exclusion criteria completed the study. There were no missing observations. Patients were on multiple anti-hypertensive drug therapies according to JNC-VIII guidelines for treatment of ISH and SDH [14]. More than 50% of patients were on single anti-hypertensive drug therapy and approximately 10% were taking combination of three drugs. None of the patient included was newly diagnosed. All patients were confirmed and stable cases of ISH and their hypertension was controlled with medications at the time of recruitment. Age and disease duration ranged from 30 to 62 years, duration 3–20 months; age 39–72 years, duration 3–12 months in SDH and ISH patients, respectively.

**Table 1:** Demographic, clinical, and biochemical characteristics of the subjects

Parameters	Group I (control) (*n* = 25)	Group II (SDH patients) (*n* = 25)	Group III (ISH patients) (*n* = 25)	*p*
Male/female number	11/14	15/10	12/13	
AGE (years)	56.00 ± 8.55	52.95 ± 12.61	55.80 ± 9.40	0.55
BMI (kg/m^2^)	25.35 ± 6.92	28.12 ± 6.52	27.37 ± 5.32	0.28
SBP (mmHg)	121.36 ± 5.15	161.75 ± 15.49	168.68 ± 7.17	< 0.001*
DBP (mmHg)	82.36 ± 3.63	102.50 ± 5.50	89.20 ± 2.55	< 0.001*
PP (mmHg)	39.00 ± 5.90	59.25 ± 13.40	79.48 ± 7.14	< 0.001*
Progranulin (ng/ml)	50.10 ± 4.70	71.36 ± 18.36	82.31 ± 16.32	< 0.001* 0.009^
TNF-α (pg/ml)	3.57 ± 1.90	19.68 ± 5.19	21.07 ± 6.32	< 0.001* 0.31^
PGRN/TNF-α ratio	23.98 ± 7.44	3.79 ± 1.33	4.32 ± 1.80	< 0.01* 0.67^

*BMI* body mass index, *SBP* systolic blood pressure, *DBP* diastolic blood pressure, *TNF-α* tumor necrosis factor alpha, *PGRN* progranulin. Results are expressed as mean ± SD; *p* significant at < 0.05 (*p value: patients vs. control; ^*p* value SDH patients vs. ISH patients)

On comparing the groups, age and BMI of healthy and patients were not significantly different (all *p* > 0.05). Since the sample size was very less, patients’ data was not subcategorized on the basis of age, sex, and BMI. All the patients and healthy subjects were overweight (average BMI, 25–29.9 kg/m^2^), but none was having dyslipidemia and on any weight reduction/hypolipidemic treatment. In this study, low TNF-α levels and high PGRN levels in healthy subjects revealed absence of low-grade inflammation and a balance between endogenous pro- and anti-inflammatory cytokines. Both progranulin and TNF-α levels were found high in SDH and ISH patients as compared to healthy subjects. High TNF-α levels depict the presence of low-grade inflammation in hypertension. Elevated PGRN levels in response to raised TNF-α levels depicts the counter regulation by PGRN to neutralize TNF-α. Moreover, PGRN/TNF-α ratio was found significantly decreased in ISH and SDH patients as compared to healthy subjects.

Among all variables in ISH patients, age was significantly associated with SBP (*r* = 0.42, *p* = 0.04) and PP (*r* = 0.43, *p =* 0.03); SBP with PGRN (*r* = 0.44, *p* = 0.03) and PP (*r* = 0.98, *p* < 0.01); PGRN/TNF-α ratio with PGRN (*r* = 0.59, *p* = 0.02), TNF-α (*r* = − 0.79, *p <* 0.01), and PP (*r* = 0.42, *p* = 0.04).

Among all variables in SDH patients, both age and BMI were not significantly correlated with any parameter like SBP, DBP, PP, PGRN, and TNF-α. SBP was correlated with PP (*r* = 0.93, *p* < 0.01), DBP (*r* = 0.53, *p* = 0.01), PGRN (*r* = 0.49, *p =* 0.02), and TNF-α (*r* = 0.46, *p* = 0.03); PP with TNF-α (*r* = 0.51, *p* = 0.02) and PGRN (*r* = 0.50, *p* = 0.02). PGRN/TNF-α ratio (*r* = 0.59, *p* = 0.04) with PGRN (*r* = 0.59, *p* = 0.04) and TNF-α (*r* = − 0.61, *p* = 0.03). No other significant association was found between remaining variables (all *p >* 0.05).

Independent and linear predictors of SBP were determined using multiple linear regression analysis. Variables which showed significant correlation with SBP were entered into multiple linear regression model to find independent predictors of SBP. Only PGRN/TNF-α ratio and PP were found as independent predictors of SBP both in SDH and ISH patients after adjustment of age, sex, BMI, and DBP (Table 2).

**Table 2:** Independent predictors of SBP

S. no.	Parameters	*Β*	*T*	p^*^
Independent predictors of SBP in SDH patients				
1	PGRN/TNF-α	0.74	6.58	< 0.01
2	PP	0.85	4.7	0.03
		SBP = 5.68 + (2.023 × PGRN/TNF) + (0.56 × PP)		
Independent predictors of SBP in ISH patients				
1	PGRN/TNF-α	0.73	2.08	0.05
2	PP	0.89	22.82	<0.01
		SBP = 93.65 + (0.73 × PGRN/TNF) + (0.89 × PP)		

*PGRN* progranulin, *TNF-α* tumor necrosis factor-alpha, *PP* pulse pressure

*p < 0.05 statistically significant

## Discussion

Low-grade inflammation imparts a crucial pathogenic role in systolic-diastolic hypertension and isolated systolic hypertension. This study had revealed an imbalance between pro-inflammatory cytokine (TNF-α) and anti-inflammatory growth factor (PGRN) in hypertensive patients. Reduced PGRN/TNF-α ratio further supports the inflammatory basis of the disease. Moreover, PGRN/TNF ratio was also found as independent predictor of SBP that ascertain the significance of inflammatory component responsible for increasing SBP.

ISH is the most common form of hypertension in those older than 65 years [17]. Lloyd-Jones et al. 2000 revealed that increasing age was significantly associated with lack of SBP control [18]. Similarly, Midha et al. 2010 found that age, BMI and smoking were significant independent risk factors of ISH [19]. Martins et al. 2002 also demonstrated an inverse relation between BMI and PP among lean versus obese subjects with ISH [20]. In the present study, age was found significantly associated with SBP in ISH patients, but BMI was not correlated with SBP neither in ISH nor in SDH patients.

TNF-α is considered a potent pro-inflammatory cytokine which plays a crucial role in the initiation and continuation of inflammation and immunity. Various studies have shown raised levels of TNF-α in cardio-vascular diseases characterized by presence of low-grade inflammation including obesity [21], diabetes [22], metabolic syndrome [23], and atherosclerosis [24]. Present study too has shown raised serum levels of TNF-α, revealing the low-grade inflammation as an inherent feature of ISH.

PGRN acts as a strong anti-inflammatory mediator by antagonizing TNF-α signaling. The anti-inflammatory effects of PGRN are exerted through inhibition of TNF receptor (TNFR)-mediated nuclear factor-κB (NF-κB) and mitogen-activated protein kinase (MAPK) signaling by competitively binding to TNFR, especially TNFR-2, which is found in bone marrow derived macrophages [25]. Many clinical studies have reported adverse levels of circulating PGRN in many diseases characterized by presence of low-grade inflammation, including metabolic syndrome [9], diabetes mellitus [26, 27], renal dysfunction [13], and atherosclerosis [28]. Xu et al. 2015 had found a marked positive correlation between serum PGRN levels with SBP and DBP in diabetic patients having microangiopathies [26]. Present study had also found raised PGRN levels in hypertensive patients than healthy subjects, and as well had revealed an association with PP.

Given that PGRN acts as a strong anti-inflammatory mediator by antagonizing TNF-α signaling, we further investigated the relationship between serum PGRN levels and TNF-α levels in patients with ISH. We found that serum PGRN levels increased with the rising of TNF-α levels. Based on these results, we speculated that PGRN, as a competitive molecule of TNF-α, is highly expressed in hypertension secondary to the increased inflammatory cytokines, particularly, TNF-α. We speculated that the ratio of PGRN/TNF-α in serum is an important factor to evaluate the inflammatory microenvironment of patient. The findings of low PGRN/TNF ratio in hypertensives as compared to healthy subjects depicted the feedback control/regulation of TNF-α by PGRN. To confirm this speculation, we studied the relationship between serum PGRN/TNF-α ratio and SBP and found that serum PGRN/TNF-α ratio was an independent predictor of SBP. These results create a link between the concept of immunity–inflammation–blood pressure¸ a sequential and vicious network.

Present study had several limitations such as small sample size, a few numbers of variables, not measured serum soluble TNF receptor 1 (TNFR1) and TNFR2 level, no differentiation of results based on age, gender, smoking, disease severity, and other risk factors/or concomitantly present diseases. The results are based on evaluation of few patients and healthy subjects which may not justify the findings based on low statistical power of the study. In many published studies, blood pressure is taken as one of the co-variables with other main variables for observing association of serum PGRN with the disease profile. This is the first study reporting the correlation of serum PGRN/TNF ratio in systolic hypertensives in comparison to systolic-diastolic hypertensive subjects. Although results may not be robust enough but provide a preliminary data for futuristic clinical studies in hypertensive patients.

## Conclusion

The findings of reduced PGRN/TNF ratio as an independent predictor of SBP, ascertain the key role of imbalance in pro- and anti-inflammatory environment in hypertension. Thus, it strengthens the link between the concept of immunity–adiposity–inflammation–blood pressure¸ a sequential and vicious network. As PGRN is widely explored anti-inflammatory marker in various diseases, its serum levels and correlation status must be further explored with regard to age, sex, BMI, duration of hypertension, various stages, severity, and types of hypertension, as well as presence of other pro-/anti-inflammatory markers and concomitant cardiovascular disease risk variables. Further, this cross-link of SBP and progranulin must be explored in longitudinal studies to generalize the findings. New Researches should be focused not only on impact of pro-inflammatory environment rather to find on a balance between pro- and anti-inflammatory status, so that new target sites could be explored for therapeutic management of hypertension.

## Data Availability

The datasets used and/or analyzed during the current study are available from the corresponding author on reasonable request. All data generated or analyzed during this study are included in this published article.

## Abbreviations

PGRN: Progranulin
TNF-α: Tumor necrosis factor-alpha
PP: Pulse pressure
SDH: Systolic-diastolic hypertension
ISH: Isolated systolic hypertension
BMI: Body mass index
